# Taxonomic and Phylogenetic Determinants of Functional Composition of Bolivian Bat Assemblages

**DOI:** 10.1371/journal.pone.0158170

**Published:** 2016-07-06

**Authors:** Luis F. Aguirre, Flavia A. Montaño-Centellas, M. Mercedes Gavilanez, Richard D. Stevens

**Affiliations:** 1 Centro de Biodiversidad y Genética, Universidad Mayor de San Simón, Cochabamba, Bolivia; 2 Department of Wildlife Ecology and Conservation, University of Florida, Gainesville, Florida, United States of America; 3 Escuela de Ciencias Biológicas y Ambientales, Facultad de Ciencias Médicas, Universidad Central del Ecuador, Quito, Ecuador; 4 Department of Natural Resources Management and the Museum of Texas Tech University, Lubbock, Texas, United States of America; Università degli Studi di Napoli Federico II, ITALY

## Abstract

Understanding diversity patterns and the potential mechanisms driving them is a fundamental goal in ecology. Examination of different dimensions of biodiversity can provide insights into the relative importance of different processes acting upon biotas to shape communities. Unfortunately, patterns of diversity are still poorly understood in hyper-diverse tropical countries. Here, we assess spatial variation of taxonomic, functional and phylogenetic diversity of bat assemblages in one of the least studied Neotropical countries, Bolivia, and determine whether changes in biodiversity are explained by the replacement of species or functional groups, or by differences in richness (i.e., gain or loss of species or functional groups). Further, we evaluate the contribution of phylogenetic and taxonomic changes in the resulting patterns of functional diversity of bats. Using well-sampled assemblages from published studies we examine noctilionoid bats at ten study sites across five ecoregions in Bolivia. Bat assemblages differed from each other in all dimensions of biodiversity considered; however, diversity patterns for each dimension were likely structured by different mechanisms. Within ecoregions, differences were largely explained by species richness, suggesting that the gain or loss of species or functional groups (as opposed to replacement) was driving dissimilarity patterns. Overall, our results suggest that whereas evolutionary processes (i.e., historical connection and dispersal routes across Bolivia) create a template of diversity patterns across the country, ecological mechanisms modify these templates, decoupling the observed patterns of functional, taxonomic and phylogenetic diversity in Bolivian bats. Our results suggests that elevation represents an important source of variability among diversity patterns for each dimension of diversity considered. Further, we found that neither phylogenetic nor taxonomic diversity can fully account for patterns of functional diversity, highlighting the need for examining different dimensions of biodiversity of bats in hyperdiverse ecosystems.

## Introduction

Elucidating spatial variation in species composition among habitat types (‘diversity patterns’ *sensu* Magurran [1]), and the potential mechanisms driving these patterns are fundamental goals of ecologists and evolutionary biologists [2]. However, spatial patterns of diversity are still poorly understood, and most of the research has historically focused only on the taxonomic dimension of biodiversity. Biodiversity, however, is a multifaceted concept, composed of multiple dimensions that are likely related to different processes driving diversity patterns [3]. Accounting only for taxonomic characteristics of species may produce an incomplete or biased view of biodiversity patterns, as it relies on the assumptions that all species are equally distinct and patterns are not sensitive to ecological and evolutionary variation among species [4]. However, species are not equivalent. Evolutionary differences between species are reflected in their phylogenetic relatedness [5]. Phylogenetic diversity measures these differences based on the time since divergence from a common ancestor [6]. In contrast, because species ecological attributes can be related to their functions within ecosystems, functional diversity measures this variability among species and provides a mechanistic link to ecosystem functioning [7]. Simultaneous examination of these different dimensions of diversity might provide deeper insights into the potential mechanisms underlying patterns of diversity and distribution. For instance, how redundant (i.e. correlated) are different dimensions of diversity or how decoupled (i.e. non-correlated) they are, might highlight the potential role of ecological and historical (i.e. geographical and evolutionary) processes driving community assembly [8, 9]. Furthermore, effectiveness of taxonomic diversity as a surrogate of biodiversity can be examined by comparing how other dimensions that include evolutionary histories and ecological functions (i.e. phylogenetic and functional diversity, respectively) relate to taxonomic diversity.

Changes in species composition among assemblages can be related to two different mechanisms: species turnover (i.e. the replacement of one species by another species) and species loss or gain causing nestedness among assemblages (i.e. richness difference) [10, 11]. The same principle can be applied to different dimensions of biodiversity by changing the unit of diversity. For example, differences in the number and identity of the functional groups can produce assemblages that differ in functional diversity due to functional richness or turnover, respectively. Surprisingly, metrics of community dissimilarity that account for species functional or phylogenetic relationships have only been developed recently [12] and no study has yet to assess the relative importance of these components in dissimilarity patterns in any Andean country.

Despite the increasing number of studies assessing multiple dimensions of biodiversity, our understanding of the processes driving spatial patterns of functional diversity in the Neotropics is still limited [3, 8, 13–18]. Here, we assess patterns of taxonomic, functional and phylogenetic diversity of bat assemblages in one of the least studied countries in the Neotropics, Bolivia [19]. We examine bat biodiversity patterns and their variation across ecoregions to gain insights into the potential role of ecological and historical processes driving biodiversity patterns. We complement this approach with an assessment of components (i.e. replacement and richness differences) of functional, taxonomic and phylogenetic dissimilarity patterns, to better understand the mechanisms acting upon bat faunas across Bolivia. Overall we expected that assemblages in the same ecoregion would be more similar to each other in all dimensions of diversity, whereas assemblages from different ecoregions will differ in functional, taxonomic and/or phylogenetic diversity. Further, because species replacement is expected to occur along ecological gradients that are sufficiently long to cause simultaneous gain and loss of species we expected this to be higher among ecoregions. On the contrary, as differences in species richness are likely to reflect smaller scale ecological processes (e.g. physical barriers) and diversity of niches available we expected number of species to be important for explaining within ecoregion dissimilarities in functional diversity among assemblages [10, 11].

Additionally, we examined the potential contribution of taxonomic and phylogenetic changes to patterns of functional diversity of bats to better understand the possible role of ecological or evolutionary processes in driving functional diversity and its variation at a country-wide scale. Because differences in phylogenetic diversity are related to differences in processes operating over longer temporal scales, we suggest that if functional diversity is strongly correlated with phylogenetic diversity, then processes shaping Bolivian bat assemblages may be largely explained by historical processes. On the other hand, if functional diversity is more related to taxonomic diversity, without a clear phylogenetic signal, this would suggest that more short-term ecological processes may explain differences among assemblages. Furthermore, if overall correlations among different dimensions of biodiversity are high, this may suggest that diversity patterns are likely the result of one or a few mechanisms driving the assemblage of ecological communities. In contrast, if low correlations are found among different dimensions of biodiversity, this would suggest that different processes are likely controlling different dimensions of biodiversity [8].

## Materials and Methods

**Fig 1:**
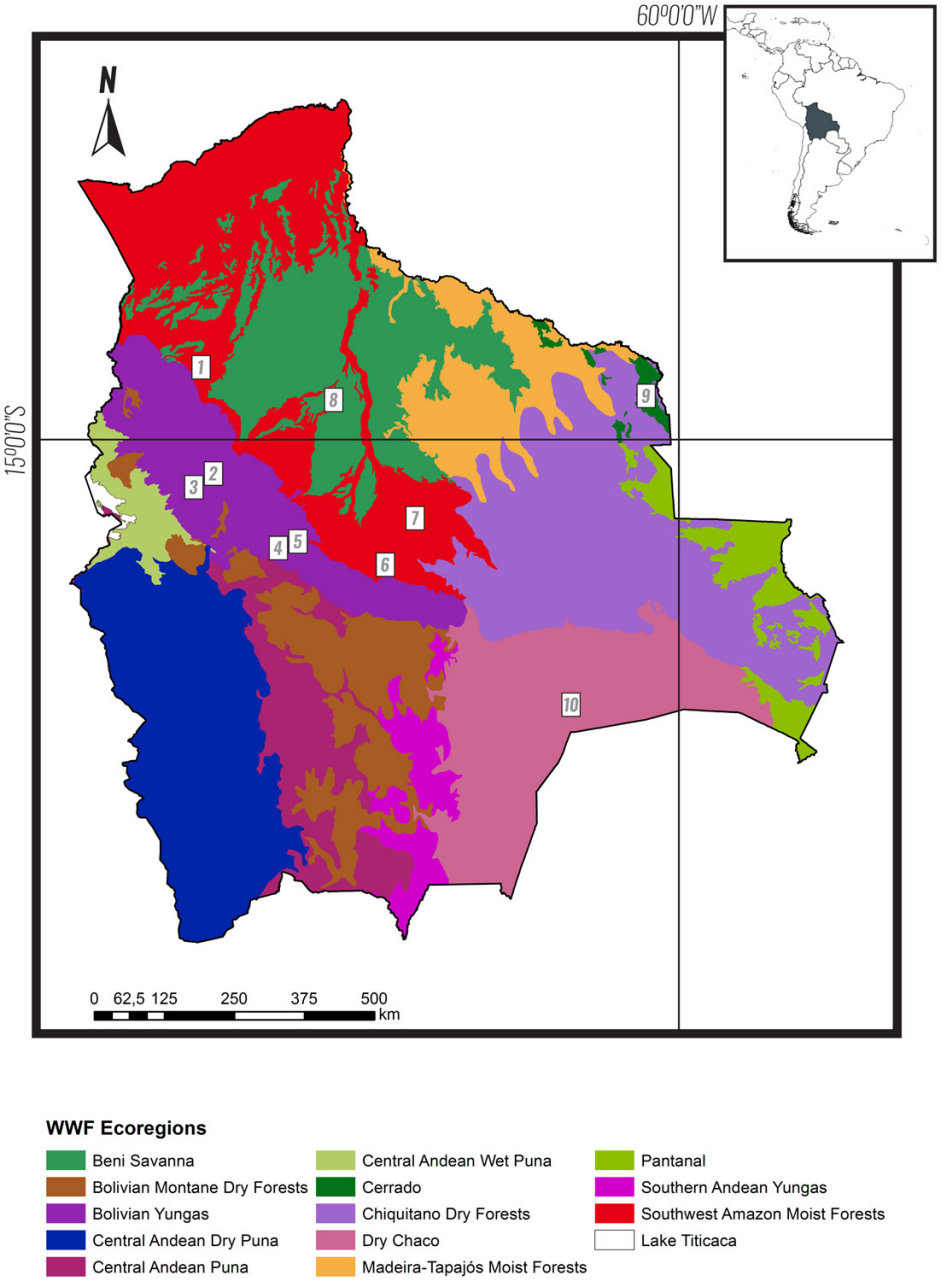
Ecoregions of Bolivia and location of studies included in our analyses. Limits of ecoregions follow the World Wildlife Fund [21]. The figure is similar but not identical to the original WWF figure, and is therefore for illustrative purposes only.

Bolivia is situated in South America between latitudes 9°38’ S and 22°53’ S and longitudes 57°26 and 69°38’ W. Its main physiographic feature is the Andean Cordillera, which crosses the country from northwest to south and is the origin of rivers belonging to three drainages: the Amazon Basin (66% of the country’s extension), the Paraná Basin (21%), and the Altiplano Basin (13%) [20]. The World Wildlife Fund of Nature recognized 13 main ecoregions in Bolivia [21](Fig 1). Seven ecoregions are located in the lowlands and range from the humid SW Amazon to the North of the country, to the Dry Chaco in the South. Three ecoregions are located on East Andean slopes, and range (North to South) from the humid Bolivian Yungas, to the Bolivian Montane Dry Forest. The last three ecoregions are located in the high mountain ranges and highlands that range from the wet Puna to the North, towards the dry Puna to the South [22].

We examined bat assemblages at ten sites across five ecoregions in Bolivia (Table 1). Data were collected from published studies that represented well-sampled assemblages, exhibiting asymptotic species accumulation curves [23–33]. To be included in our study, data should represent spatially delimited local communities (i.e. be collected at one location, Stevens and Gavilanez 2015). At each site, bats were sampled using ground-level mist nets, thus we focused our analyses on a subset of the species registered in each community, the assemblage of Noctilionoidea. The superfamily Noctilionoidea includes families Phyllostomidae, Noctilionidae and Mormoopidae, all of which can be reliably surveyed with mist nets [34]. Furthermore, Noctilionoidea is a monophyletic [35], species rich and phenotypically diverse group, making it an ideal subject for analyses of biodiversity. Hereafter we will simply use the term ‘bats’ to refer to the Noctilionoidea species included in our analyses. To explore potential biases of sampling effort in our analyses, we tested for correlations between sampling effort and species richness and number of captured individuals across sites. For comparison, sampling effort was calculated in standard mist-net hours (a 12m mist net open for one hour; *sensu* Ralph [36]). As we found no relationships between these variables, our measurements of diversity are comparable among sites even though sampling effort varied among communities (S1 Fig).

**Table 1:** Bat assemblages included in our analyses. Data sets were extracted from published and unpublished sources. Numbers in the first column correspond to locations shown in Fig 1. Effort is presented in mist net hours. N° of individuals refer to the number of captured bats in the Noctilionoidea superfamily only. SR = Species richness of noctilionoid bats, FR = Functional richness, the number of functional guilds in the assemblage, FR Chao = estimated functional richness using Chao non-parametric estimate.

N°	Site	Ecoregion	Mean elevation (m asl)	Effort	N° individuals	SR	FR	FR Chao	References
1	Pie de Monte—Terán	Southwestern Amazonia	185	33000	357	37	8	8.00	[26]
2	Pie de Monte -Flores	Bolivian Yungas	650	22920	571	31	7	7.00	[29]
3	Yungas Medio	Bolivian Yungas	1520	153012	1579	21	5	5.20	[30]
4	Yungas Alto	Bolivian Yungas	3000	34174	119	7	4		[23]
5	Pie de Monte -Vargas	Bolivian Yungas	500	50795	2542	34	6	6.000	[31]
6	Amazónico-Ichilo	Southwestern Amazonia	220	97889	272	21	8	8.0	[32]
7	Madre de Dios	Southwestern Amazonia	185	45820	804	20	7	7.14	[27]
8	Sabana Inundable	Beni Savannas	170	382269	594	26	9	9.00	[24, 25]
9	Cerrado	Cerrado	600	177120	383	25	9	9.11	[33]
10	Chaco	Dry Chaco	300	21459	163	9	7	7.14	[28]

Species names for all sites were standardized using those found in Wilson and Reeder [37]. We considered functional diversity as the diversity of ways bats contribute to important ecosystem processes, such as production and immobilization, and important ecosystem services such as seed dissemination and flower pollination, via their foraging behavior [3, 38]. Therefore, species within assemblages were assigned to one functional group based on their foraging guilds following Kalko [39], Kalko et al. [40], Sampaio et al. [41] and Aguirre [42]. Functional groups represented in our study were highly cluttered space gleaning canopy frugivores, highly cluttered space gleaning understory frugivores, highly cluttered space gleaning nectarivores, highly cluttered space gleaning carnivores, highly cluttered space gleaning omnivores, highly cluttered space gleaning piscivores, highly cluttered space gleaning sanguinivores, highly cluttered space gleaning insectivores, highly cluttered space aerial insectivores and background cluttered space aerial insectivores. We used the richness of functional groups and the inverse of Simpsons’ index [1] calculated on the number of species per functional group to characterize functional diversity for each assemblage, and contrasted them with the Chao non-parametric estimator [43]. To assess if differences in functional richness are determined by sampling abundances (i.e. the number of individuals per functional group) we rarefied to 100 individuals and examined results. Additionally, we created a functional rank-richness distribution curve for each site. Typically, rank distributions order species from the most abundant to the least abundant and are referred to as rank-abundance distributions [1]. We ranked functional groups from those with the greatest number of species to those with the least when constructing functional rank-richness distributions.

We used a bat supertree by Bininda-Emonds et al. [44] to summarize phylogenetic relationships among species. This tree was pruned to the subset of Noctilionoidae species found across the 10 sites (S2 Fig). In some cases taxonomic conflicts occurred, in particular when subspecies were elevated to the species level. In all cases, the sister taxon (originally the parent species of the particular subspecies) did not occur across the sampled sites and we used this taxon to represent the elevated species when calculating phylogenetic distance.

We examined differences in taxonomic, functional and phylogenetic diversity among assemblages. Pairwise differences in taxonomic diversity between sites were estimated using Bray-Curtis distances, calculated on relative abundance of individuals per species at each site. Differences in functional composition were also based on Bray-Curtis distances but calculated on relative number of individuals per functional group at each site. Bray-Curtis distances were calculated using package *ecoDist* [45] in R [46]. Differences in phylogenetic diversity were calculated based on weighted UniFrac distances, with a controlling parameter alpha of 0.5, using functions in package *GUniFrac* [47]. We used the unweighted pair-group method with arithmetic mean (UPGMA) algorithm to cluster sites based on distance matrices (taxonomic, functional and phylogenetic), using package *cluster* [48]. We characterized fit of the data to the bifurcating phenogram produced by UPGMA based on their agglomerative coefficients and cophenetic correlation coefficients [49]. Finally, to complement our analyses we assessed if dissimilarity between sites in terms of taxonomic, functional or phylogenetic diversity is mostly explained by replacement (i.e. species turnover; β_repl_) or richness differences (loss or gain of species or functional groups; β_repl_) using functions in package BAT [12].

We were also interested in examining the relative contributions of differences in taxonomic and phylogenetic diversity to variation among sites regarding functional diversity. For this, we used Multiple Regression on Distance Matrices (MRM) performed in the package *ecodist* [45], with the functional distance matrix as response variable and taxonomic and phylogenetic distance matrices as explanatory variables. Statistical significance of the MRM model and of each explanatory matrix was obtained by 10000 permutations, permuting the response matrix while holding the explanatory matrices constant [45, 50]. Finally, to further explore the relative contribution of taxonomic and phylogenetic differences on functional diversity patterns, we followed Chevan and Shuterland [51] hierarchical partition method implemented in the package *hier*.*part* [52]. Hierarchical partition explores the relative contribution of each explanatory matrix by comparing the goodness of fit of all possible MRM models ordered in growing complexity: from the one with no explanatory matrix (R^2^ = 0) to the full model with both explanatory matrices. All statistical analyses were conducted using R [46].

## Results

**Fig 2:**
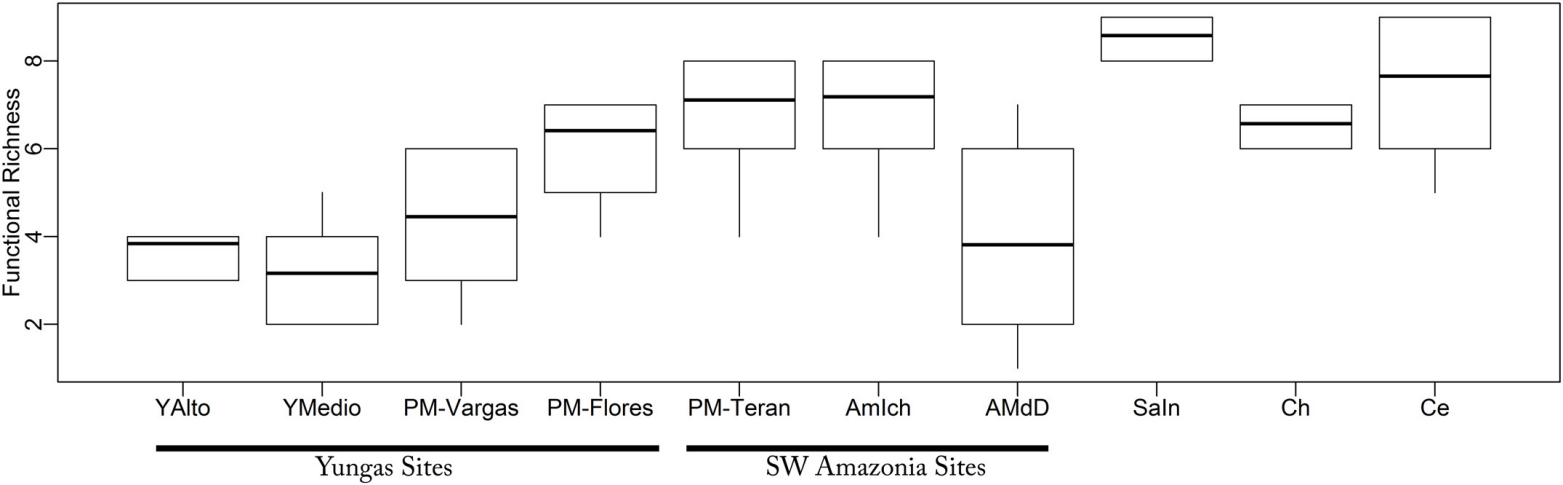
Results of the rarefaction procedure, for 100 individuals and 1000 runs for functional richness of bats in ten study sites across Bolivia. Sites are YAlto = Yungas Alto, YMedio = Yungas Medio, PM-Vargas = Pie de Monte—Vargas, PM-Teran = Pie de Monte-Terán, AmIch = Amazonica Ichilo, AMdD = Amazonia Madre de Dios, SaIn = Sabana Inundable, Ch = Chaco, Ce = Cerrado. Sites belonging to the same ecoregion are underlined.

Sixty of the 84 species of the super-family Noctilionoidea known for Bolivia were present across our study sites, including two species in the family Noctilionidae, one in Mormoopidae and 57 species in Phyllostomidae. Species richness of bats per assemblage ranged from 37 species in Pie de Monte-Terán, in the SW Amazonia ecoregion, to only seven species in Yungas Alto, a high elevation site in the Bolivian Yungas ecoregion (Table 1). The number of functional groups per assemblage ranged from 4 to 9, and functional diversity ranged from D_inv_ = 2.58 for Yungas Alto to 6.63 for Sabana Inundable in the Beni Savanna ecoregion. Both rarefaction analyses and Chao estimators suggested that Sabana Inundable was the most functionally rich assemblage whereas the three SW Amazonia assemblages as well as the Chaco and the Cerrado assemblages had similar functional richness. Yungas assemblages were the least diverse in terms of functional richness (Fig 2, Table 1).

**Fig 3:**
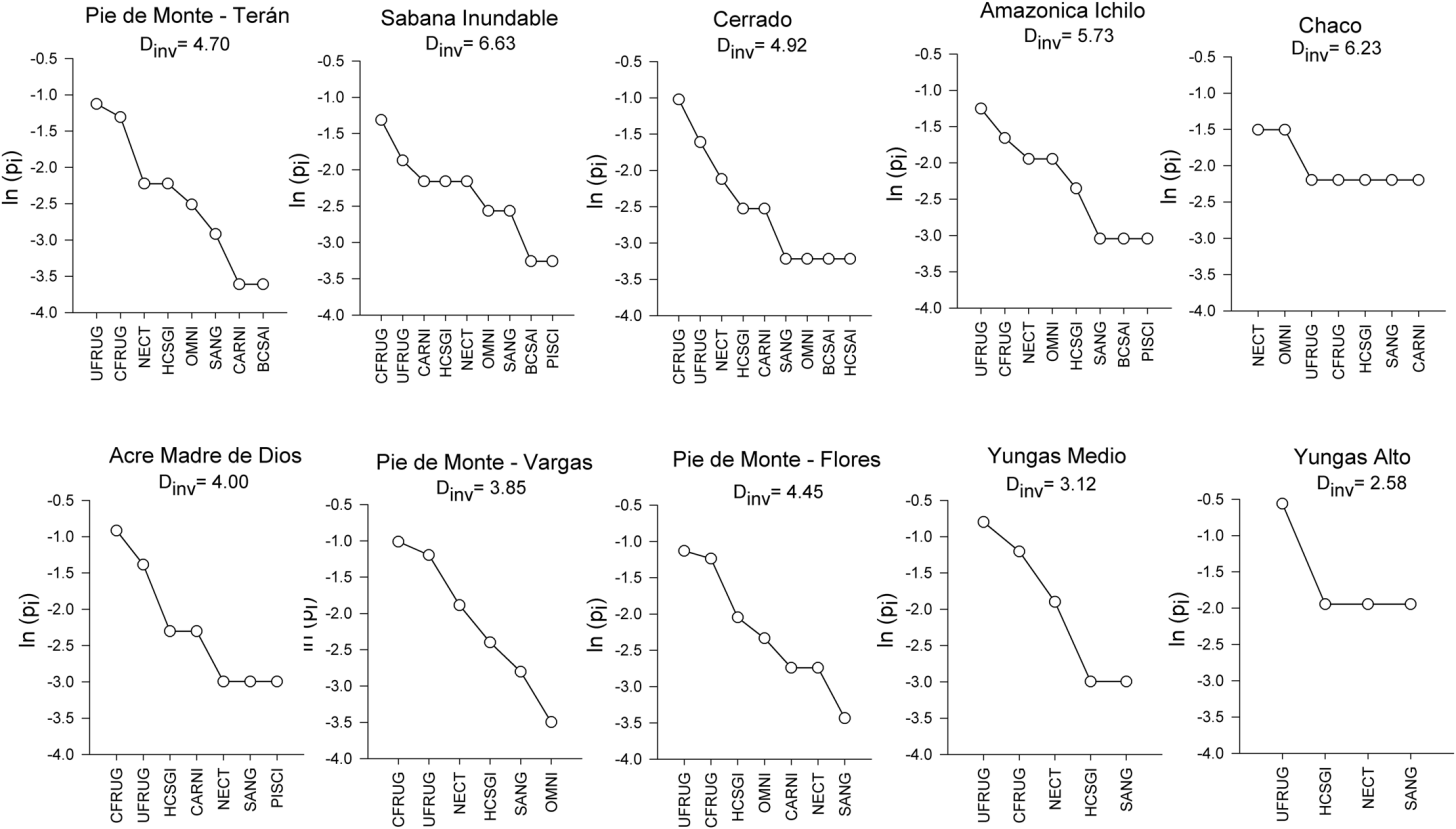
Functional rank-richness distribution curves for bat assemblages at ten locations in Bolivia. Curves were constructed for each study site by ranking functional groups from those with the greatest number of species to those with the least number of species. Functional groups are: CFRUG = Canopy frugivores, UFRUG = understory frugivores, NECT = nectarivores, CARN = carnivores, OMN = omnivores, PISCI = piscivores, SANG = sanguinivores, HCSGI = highly cluttered space gleaning insectivores, HCSAI = highly cluttered space aerial insectivores and BCSAI = background cluttered space aerial insectivores.

Differences in the species sorting among foraging guilds created communities with differing functional evenness (Fig 3). Thus assemblages with few foraging guilds but high evenness showed high values of functional diversity (e.g. Dry Chaco site) whereas assemblages with a large number of functional groups but low evenness exhibited low functional diversity (e.g. Cerrado site). As expected, most of the assemblages were highly dominated by phyllostomid frugivores, with canopy and understory frugivores being the most species rich guilds. However, two assemblages showed a different pattern. Canopy frugivores were notably missing from the high Andean site (Yungas Alto), with understory frugivores being the dominant guild. The Chaco assemblage was the only assemblage dominated by nectarivores and omnivores.

**Fig 4:**
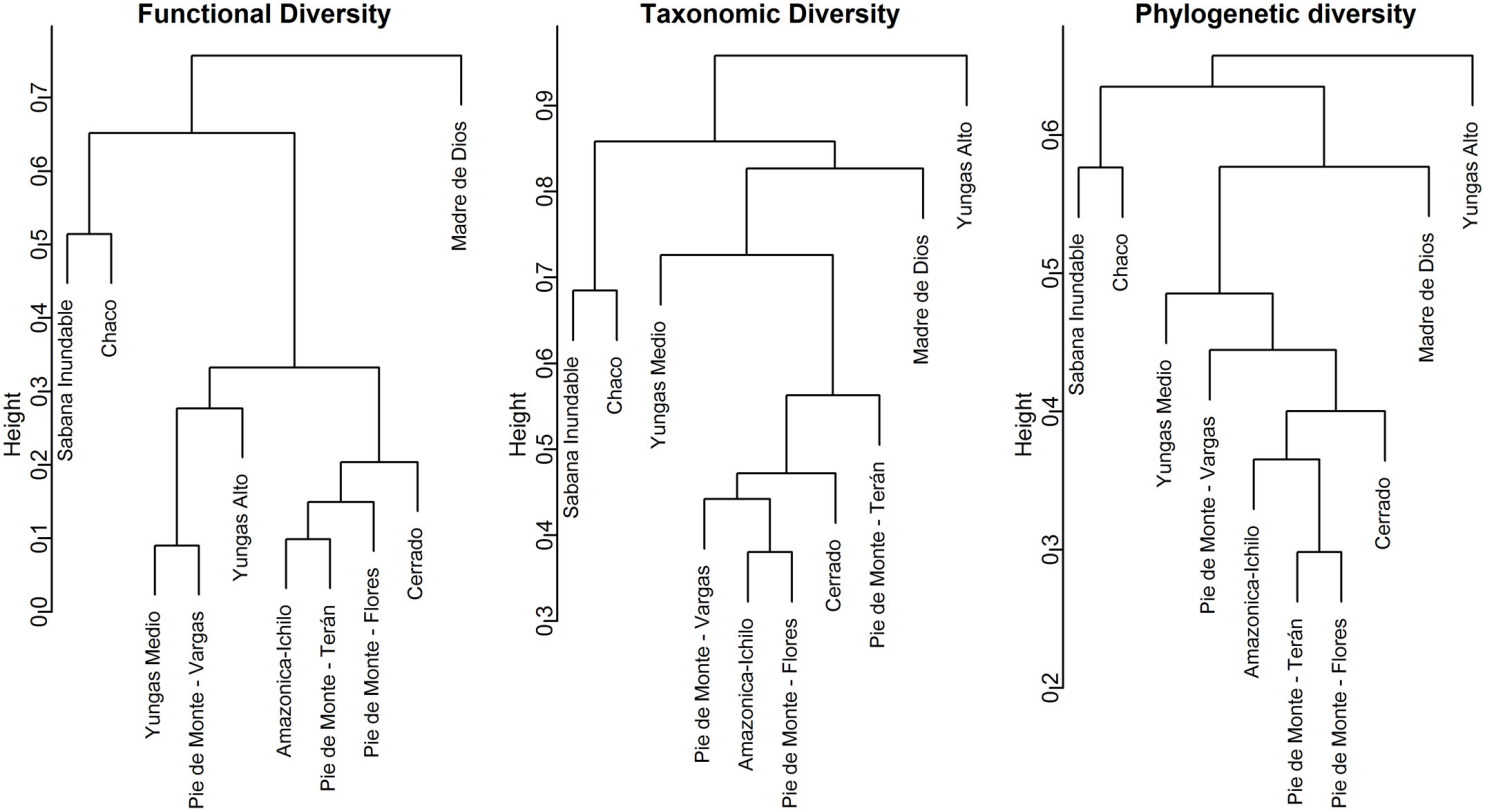
UPGMA clustering dendrograms for (A) functional, (B) phylogenetic and (C) taxonomic diversity of bats in ten assemblages across ecoregions in Bolivia. UPGMAs are calculated on relative abundance data and based on Sorensen’s dissimilarity indexes for functional and taxonomic diversity, and on the UniFrac metric for phylogenetic diversity.

Although variable across sites, functional diversity was related to differences among ecoregions (Fig 4). Functional distances among assemblages varied from 0.09 to 0.96 (mean ± SD = 0.49 ± 0.24). The cluster used to depict differences among study sites accounted for 64% of the differences among assemblages (agglomerative coefficient = 0.64, cophenetic correlation coefficient = 0.92; Table 2). When based on functional diversity, Andean assemblages within the Bolivian Yungas ecoregion clustered together. However, and more interestingly, the northern site within the Yungas province (Pie de Monte-Flores) clustered with two sites in the SW Amazonia ecoregion. The third SW Amazonia site (Madre de Dios) was separated from the rest and constituted a single site clade within the dendrogram (Fig 4). Similarly interesting was the clustering of the Cerrado site with the humid SW Amazonia assemblages, and the clustering of Sabana Inundable and Chaco, two sites of different ecoregions (Beni Savannas and Dry Chaco, respectively).

**Table 2:** Fit of cluster algorithms to distance matrices of functional, taxonomic and phylogenetic diversity, as explained by the cophenetic correlation and the agglomerative coefficient.

Dendrogram	Cophenetic Correlation	Agglomerative Coefficient
Functional diversity	0.923	0.640
Taxonomic diversity	0.935	0.361
Phylogenetic diversity	0.913	0.288

Dendrograms based on phylogenetic and taxonomic composition were substantially different than those for functional composition showing the unique variation reflected by these different dimensions of biodiversity (Fig 4, Table 2). Taxonomic distances among assemblages ranged from 0.38 to 0.99 (mean ± SD = 0.77 ± 0.18) whereas phylogenetic distances were lower, varying between 0.30 and 0.76 (mean ± SD = 0.56 ± 0.11). The phylogenetic diversity dendrogram (Fig 4) was more similar to that based on taxonomic diversity than that based on functional composition, reflecting the phylogenetic signal contained in taxonomic information for bats. Both of these perspectives indicated the uniqueness of Sabana Inundable and Chaco sites relative to the rest, separating them into one clade. Similarly, both taxonomic and phylogenetic dendrograms separated the high elevation site (Yungas alto) from the rest and retained the large separation of Madre de Dios from the other SW Amazonia sites observed in the functional diversity dendrogram. Overall, Andean sites from the Bolivian Yungas ecoregion were clustered together based on taxonomic and phylogenetic diversity, suggesting that these assemblages share several species. However, the mid elevation site (Yungas medio) was more distant to the lower elevation sites from both phylogenetic and taxonomic perspectives.

**Fig 5:**
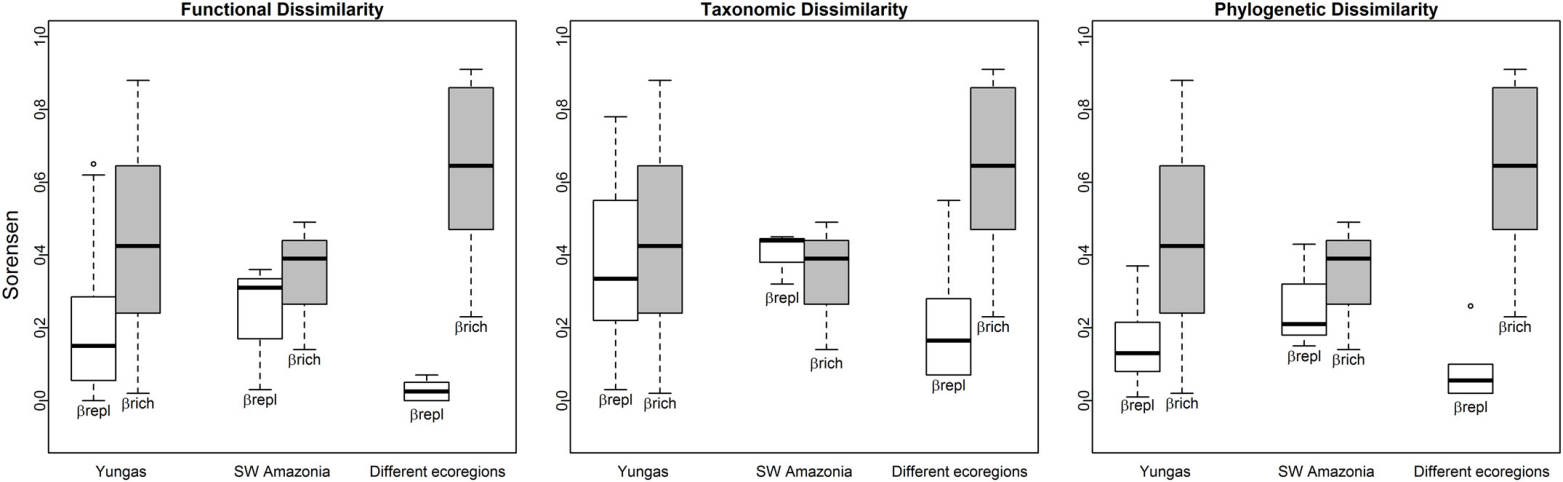
Summary of the relative importance of species replacement (β_repl_) and richness differences (β_rich_) as components of dissimilarity between assemblages from the same ecoregion (Yungas and SW Amazonia) and from sites of different ecoregions. Pair-wise dissimilarity components are summarized in boxplots for functional, taxonomic and phylogenetic dissimilarities separately. Dissimilarity values used for calculations are presented in S1 Table.

Most differences in functional diversity among assemblages were explained by loss or gain of functional groups (β_rich_) rather than functional group replacement (β_repl_; Fig 5). Although more variable, the importance of functional richness among assemblages from different ecoregions was even higher than that among assemblages from the same ecoregion. Phylogenetic dissimilarity followed a similar pattern. It was largely explained by loss or gain of species (β_rich_) rather than species replacement when assemblages belong to the different ecoregions. However, this was more evident among Yungas sites (where β_repl_ ranged between 0.02 and 0.26, and β_rich_ ranged between 0.23 and 0.91) than among SW Amazonian sites (where β_repl_ ranged between 0.20 and 0.14, but β_rich_ ranged only between 0.14 and 0.49), when comparing sites within the same ecoregions. Taxonomic dissimilarity components were slightly different from those of phylogenetic or functional dissimilarities. Overall, replacement of species (β_repl_) of sites within the same ecoregion was equally important than species loss/ gain (β_rich_) for assemblage dissimilarities when comparing sites within the same ecoregion (β_repl_ ranged from 0.07 to 0.55 for Yungas sites and from 0.32 and 0.45 for SW Amazonian sites, Fig 5). However, differences of the Yungas Alto site with other Yungas sites was largely explained by species richness (β_rich_) (S1 Table).

The MRM model explained a fair amount of variation in distances of functional diversity among sites (R^2^ = 0.52, P = 0.002). The model indicated that phylogenetic diversity contributed significantly to variation in functional diversity, but taxonomic diversity did not (Table 3). Taxonomic distances among assemblages were a poorer predictor of their functional differences than phylogenetic distances, which accounted for more than 60% of the variation explained by the model (Table 3).

**Table 3:** Results of multiple regression on distance matrices (MRM) of functional diversity as a function of taxonomic and phylogenetic diversity of Bolivian bats. MRM was calculated using a permutation method with the functional dissimilarity matrix used as the dependent matrix, and taxonomic distance and phylogenetic distance were independent matrices (F = 22.64, P = 0.002). The percentage of explained variation due to ‘pure’ effect corresponds to the contribution of each explanatory matrix to the model, as calculated by a hierarchical partitioning.

Model parameters	Coefficient	*P* value	R^2^ of the entire model	Model significance (*P* value)	Percentage of explained variation due to 'pure' effect
Intercept	-0.375	0.47	0.52	0.002	
Taxonomic distance	0.065	0.87			39.7
Phylogenetic distance	1.447	0.04			60.3

## Discussion

Bat assemblages differed from each other in all dimensions of biodiversity considered; however variation in each dimension exhibited different patterns, which suggests different structuring mechanisms may be responsible for the observed patterns of diversity. Altogether, our results suggest that both historical (i.e. historical connection and dispersal routes across Bolivia) and contemporary (such as habitat features) mechanisms are likely important in shaping bat assemblages in Bolivia. Whereas historical processes create a template of diversity patterns across the country, ecological processes modify these patterns, decoupling the observed patterns of functional, taxonomic and phylogenetic diversity in Bolivian bats.

Despite the strong phylogenetic signal of diet and foraging attributes in Neotropical bat species [16], phylogenetic diversity accounted for about 60% of the explained variation in functional diversity of bats across assemblages. The remaining 40% of the variation was explained by taxonomic differences–when the relative contribution of phylogenetic distances was controlled for. Thus, neither phylogenetic nor taxonomic diversity patterns can fully account for the functional diversity present in the sample sites, highlighting the need of examining different dimensions of biodiversity of bats across hyperdiverse ecosystems, as those present in Bolivia.

Two ecoregions were represented by more than one study sites in our data set: the Bolivian Yungas (4 sites) and SW Amazonia (3 sites). In terms of functional diversity, bat assemblages within the same ecoregion were more similar to each other than assemblages from other ecoregions. This was particularly evident among the Bolivian Yungas sites, suggesting convergent functional structure in montane regions. However Yungas assemblages differed from each other in phylogenetic and taxonomic diversity. In contrast, SW Amazonia sites showed a more consistent clustering across dimensions of biodiversity. The most eastern SW Amazonian site (Madre de Dios) formed a separate cluster, with unique functional, taxonomic and phylogenetic diversity, whereas the western SW Amazonian sites were more similar to Andean assemblages in all three dimensions.

Functional similarities across Yungas sites can be better explained when examining the functional richness rank curves. A comparison of the four sites in the Bolivian Yungas ecoregion (Yungas Alto, Yungas Medio, Pie de Monte—Vargas and Pie de Monte—Flores) suggests that there is a simplification of functional groups with an increase in elevation (S3 Fig). Both lower elevation sites (Pie de Monte—Flores and Pie de Monte—Vargas) are species rich and have similarly high functional diversity (Fig 2 and Table 1). However, the mid elevation site (Yungas Medio) lacks large carnivore and omnivore species found at lower elevations, and the higher elevation site (Yungas Alto) not only lacks these, but all species in the canopy frugivore group. This results are supported by our partitioning of dissimilarity into its components, where functional group richness (i.e loss or gain) largely explained differences in functional diversity among Yungas sites. The lack of canopy frugivores in Yungas Alto is not surprising as the height of the overall canopy layer diminishes with elevation [53] and virtually no clear forest stratification can be found in cloud forests in the Andes [54]. Nevertheless, when either phylogenetic or taxonomic diversity dimensions are considered, Yungas Alto constitutes a separate group of sites, suggesting it might differ both in terms of species identity as well as the origin of these species.

Similarly, the mid elevation assemblage Yungas medio was separated from Pie de Monte assemblages in both taxonomic and phylogenetic dimensions, whereas these latter were clustered with the SW Amazonian assemblages suggesting the inclusion of several lowland species that were potentially able to inhabit mountain foothills (Fig 3). This result might reflect the loss of lowland species in bat assemblages with increased elevation, as has been described for several other taxa [4, 17, 55]. Higher elevation communities might be composed almost entirely of Andean taxa as lowland species fail to disperse to these high elevation ranges [56]. For instance, montane species like *Anoura geoffroyi*, *Sturnira erythromos* and *S*. *oporaphilum* become numerically dominant in Yungas medio and Yungas Alto assemblages, whereas the relative abundance of other nectarivores and larger frugivores decreases. Again, the high relevance of species richness (and low importance of species replacement) on taxonomic and phylogenetic dissimilarities among Yungas assemblages further supports this finding. Although preliminary, our results highlight the need for further examination of functional beta diversity patterns of bats along elevation gradients in the Bolivian Andes.

Three ecoregions included in this study were represented by a single assemblage: Cerrado, Beni Savannas and Dry Chaco. In spite of their ecological differences and geographical distances (Fig 1), the Beni Savannas assemblage (Sabana Inundable) and the Dry Chaco assemblage (Chaco) formed a distinct cluster in all three dimensions of diversity. This result is consistent with the hypothesis of an ancient contact among Atlantic Forest and the Amazon supported by phylogeographic and molecular studies in several taxa [57, 58]. Suggested routes for faunal movement across the Atlantic Forest and the Amazon cross both our Chaco and Sabana Inundable sites. This is further supported by our partitioning of dissimilarity components suggesting low replacement of clades and functional groups between Chaco and Sabana Inundable. Similarity among these sites can also be explained given a closer examination of component species. First, the genus *Sturnira* is represented by a single species at both sites: *S*. *lilium*. The genus *Sturnira* is rooted in the Andes and *S*. *lilium* is the only species common in the lowlands belonging to one of the two subclades described by Velazco and Patterson (2013) present in our data set. All other *Sturnira* bats in our study belong to the second subclade found in their study, a montane clade. Furthermore, although *S*. *lilium* is present in our montane sites, montane individuals are likely part of a new undescribed species as suggested by recent molecular data [59], thus the phylogenetic distances among the Savanna-Chaco cluster and the other sites might be even larger than suggested by our study. Second, our Savanna and Chaco assemblages are the only two where *Phyllostomus discolor*, a large nectarivore, was recorded (20 captures in Sabana Inundable and 25 in Chaco). This species diverged earlier from the other *Phyllostomus* species in our data set (S2 Fig) and represents the only nectarivore *Phyllostomus*. Third, these two sites, along with the Cerrado assemblage, were the only ones lacking *Carollia brevicauda*, an abundant species in Andean and Amazon communities.

The third ecoregion represented by a single assemblage, Cerrado, clustered among the low elevation montane sites (Pie de Monte) for all three dimensions of diversity. This result was puzzling at first, given the large geographical distance and strong ecological differences among these sites. The Cerrado constitutes a mosaic of savannas and semi-deciduous forests, whereas Pie de Monte is dominated by evergreen hyper humid forest [22]. However, similarities among these assemblages can be partially explained when examining their shared species. For instance, our Cerrado site falls into the connection routes that might have permitted the exchange of forest species between regions that are currently isolated by more open habitats, as suggested by various studies [57, 58]. Some of the species in the Cerrado assemblage are common and widely distributed among SW Amazonian and Pie de Monte sites (e.g. *Artibeus planirostris*, *A*. *obscurus*, *Phylloderma stenops*, *Mesophylla maconelli*). Additionally, several netting sites in the Cerrado study were placed in the plateau of the Serrania de Huanchaca (600–900 m asl), a similar elevational range of the Pie de Monte sites. As a result, although several frequent species in the Cerrado site are common in the Beni Savannas site (e.g. *Phyllostomus elongatus*, *Tonatia silvicola*, *Trachops cirrhosus*), dominant species in the Cerrado assemblage are also frequent in the Pie de Monte sites (e.g. *Artibeus lituratus*, *Carollia perspicillata*, *Platyrrhinus incarum*, *Uroderma bilobatum*). Furthermore, some of the rare species in the Cerrado assemblage are shared only with Pie de Monte sites, such as *Chiroderma villosum* and *Lonchophylla thomasi*. Both sharing numerically dominant species and rare species might explain the similarity patterns between Cerrado and Pie de Monte bat assemblages in all dimensions of diversity.

Overall our results highlight the importance of examining multiple dimensions of biodiversity when exploring diversity patterns at large scales, as different patterns might contain non-redundant information about the processes shaping diversity. Furthermore, they offer an example of the additional insights provided by partitioning dissimilarity into its components. For Bolivian bat assemblages, our study suggests that evolutionary processes have shaped phylogenetic diversity and remain the most important explanation of current diversity patterns at a nationwide scale. However, ecological processes modify these templates to create differences, for example the convergence of functional roles among montane species in the montane sites of the Bolivian Yungas. Our analyses underlie the need for more detailed inspection of different aspects of bat diversity to disentangle the driving forces shaping these communities within ecoregions. As bat research in Bolivia continues to grow, we expect to be able to include more information to further analyze this topic.

## Supporting Information

S1 Fig Relationship between sampling effort in mist net hours (MNH) and the number of species and number of individual Noctilionoidea bats captured at ten study sites in Bolivia. (DOCX)

S2 Fig Phylogenetic relationships among all Noctilionoidae species included in this study. (DOCX)

S3 Fig Species richness (SRich) and functional richness (FRich) of Bolivian bat assemblages across elevations. Note the decay in functional and taxonomic richness along elevation in the Bolivian Yungas sites. Elevation is presented in m above sea level.
(DOCX)

S1 Table Components of dissimilarity between bat assemblages from ten study sites across Bolivia. Components are: βrepl = dissimilarity due to replacement of species or functional groups and βrich = dissimilarity due to differences in species or functional group richness (i.e. loss or gain of species or functional groups).
(DOCX)
