# Supplementary material for: Taxonomic and Phylogenetic Determinants of Functional Composition of Bolivian Bat Assemblages
Source: PLoS One. 2016 Jul 6;11(7):e0158170. doi: 10.1371/journal.pone.0158170 (PMC4934923; doi:10.1371/journal.pone.0158170)

**S1 Fig. Relationship between sampling effort in mist net hours (MNH) and the number of species and number of individual Noctilionoidae bats captured at ten study sites in Bolivia**. No significant correlation was found between variables (*r =* 0.11, *P =* 0.75 for species richness and *r =* 0.01 and *P =* 0.97 for number of captured individuals).


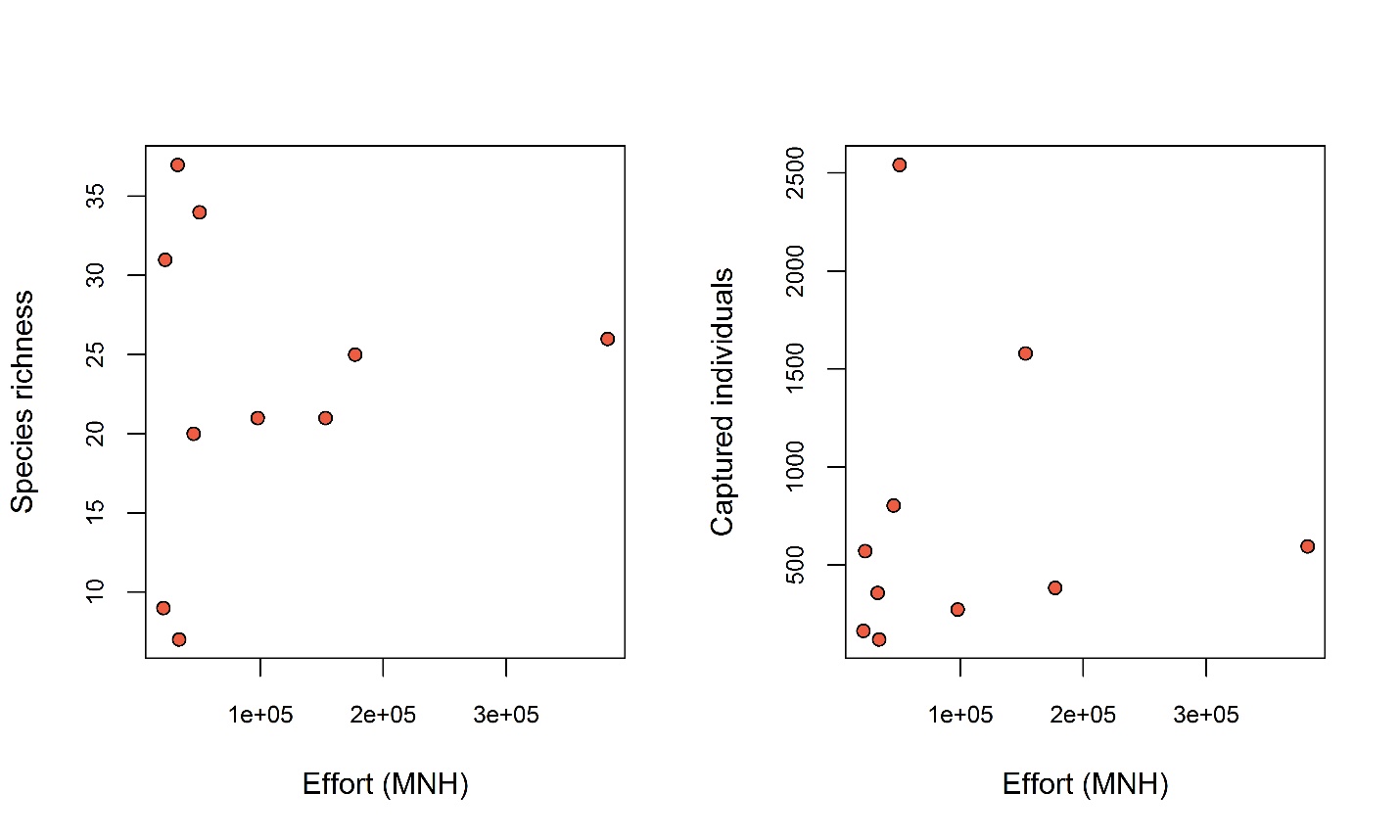

Supplement: S1 Fig — (DOCX) [file pone.0158170.s001.docx]
