# Supplementary figures and images for: Taxonomic and Phylogenetic Determinants of Functional Composition of Bolivian Bat Assemblages
Source: PLoS One. 2016 Jul 6;11(7):e0158170. doi: 10.1371/journal.pone.0158170 (PMC4934923; doi:10.1371/journal.pone.0158170)

**S2 Fig. Phylogenetic relationships among all Noctilionoidae species included in this study.**


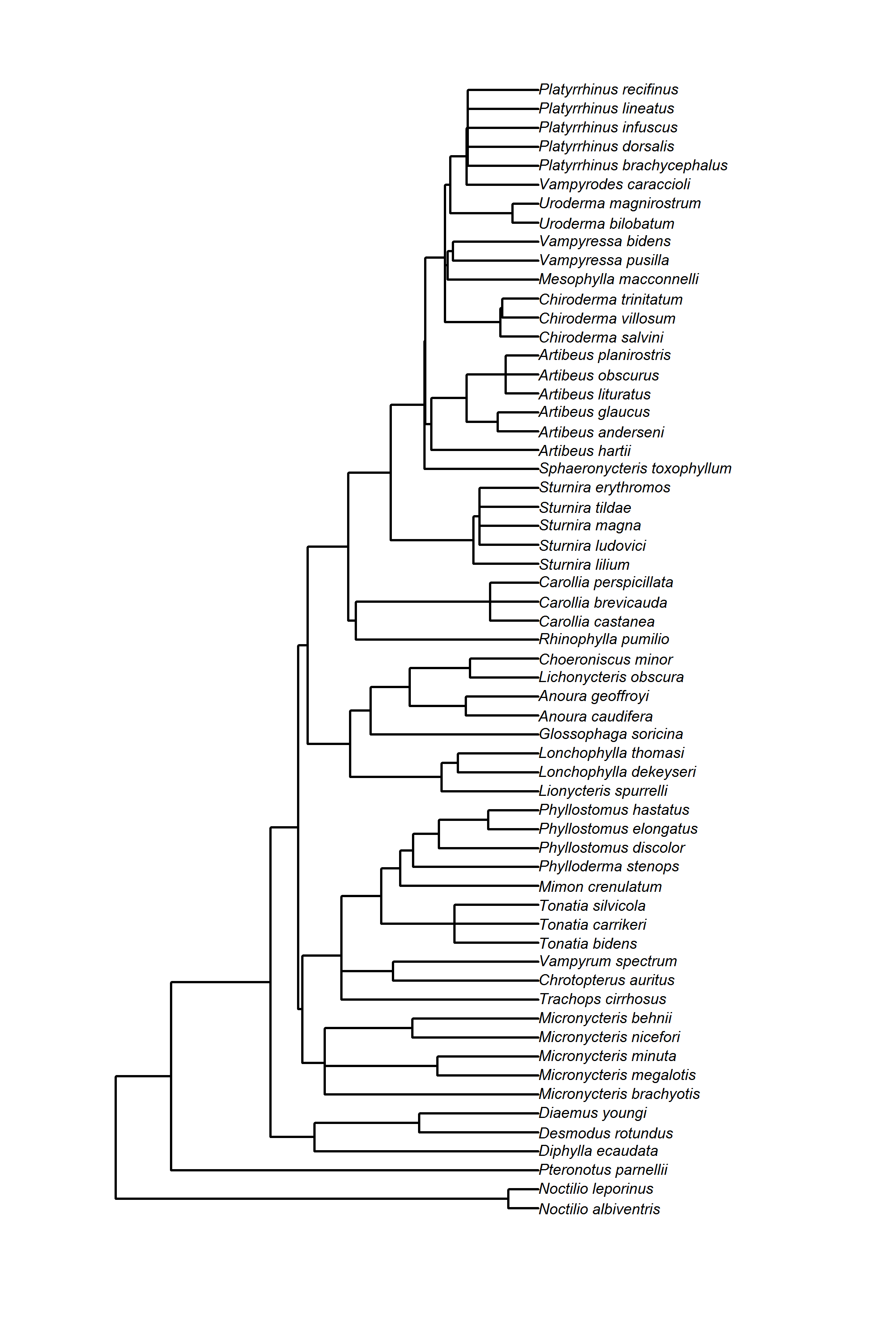

Supplement: S2 Fig — (DOCX) [file pone.0158170.s002.docx]
