# Supplementary material for: Taxonomic and Phylogenetic Determinants of Functional Composition of Bolivian Bat Assemblages
Source: PLoS One. 2016 Jul 6;11(7):e0158170. doi: 10.1371/journal.pone.0158170 (PMC4934923; doi:10.1371/journal.pone.0158170)

**S3 Fig. Species richness (SRich) and functional richness (FRich) of Bolivian bat assemblages across elevations. Note the decay in functional and taxonomic richness along elevation in the Bolivian Yungas sites. Elevation is presented in m above sea level.**


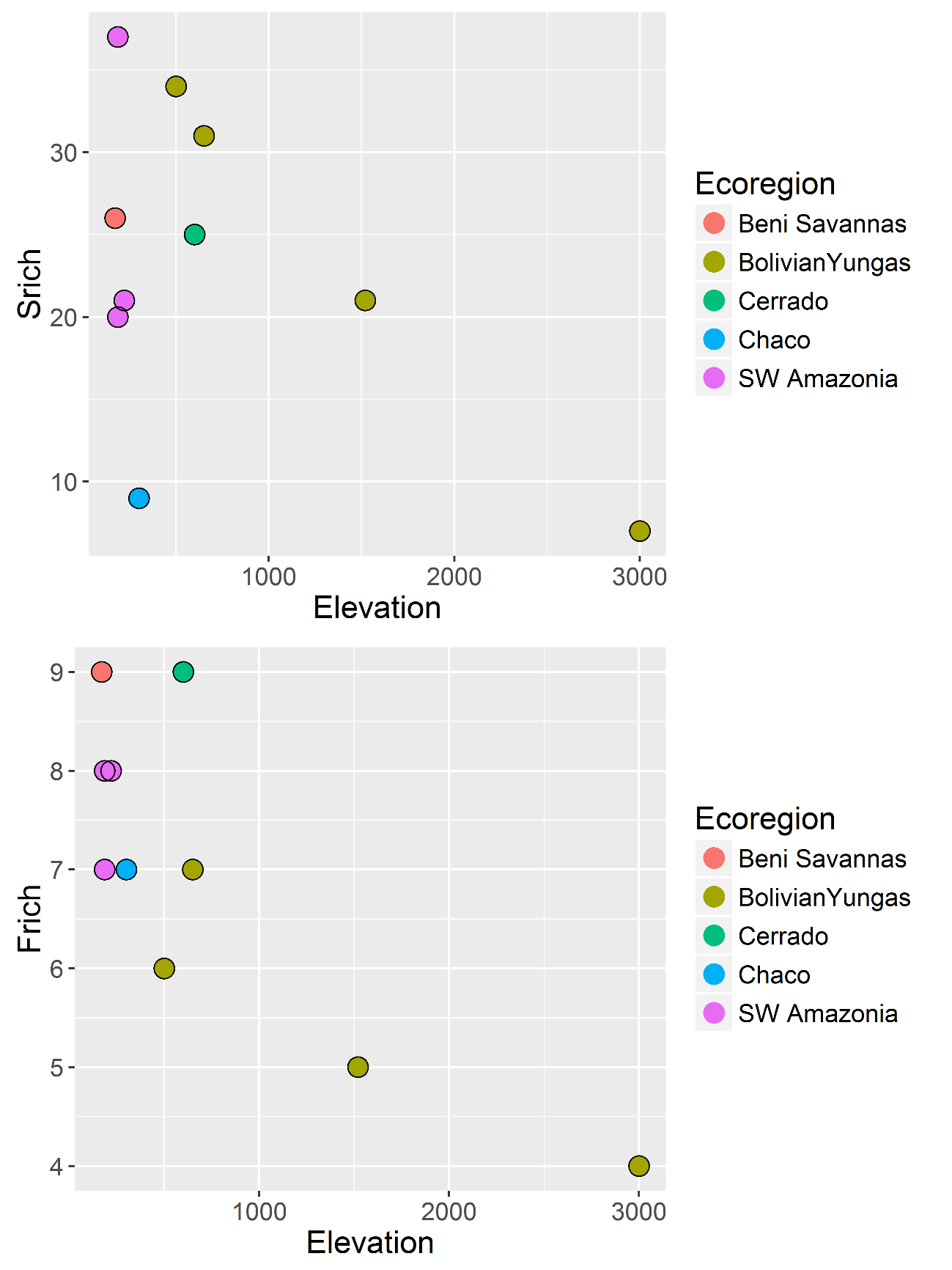

Supplement: S3 Fig — Note the decay in functional and taxonomic richness along elevation in the Bolivian Yungas sites. Elevation is presented in m above sea level. (DOCX) [file pone.0158170.s003.docx]
