# Supplementary material for: Taxonomic and Phylogenetic Determinants of Functional Composition of Bolivian Bat Assemblages
Source: PLoS One. 2016 Jul 6;11(7):e0158170. doi: 10.1371/journal.pone.0158170 (PMC4934923; doi:10.1371/journal.pone.0158170)
Supplement: S1 Table — Components are: βrepl = dissimilarity due to replacement of species or functional groups and βrich = dissimilarity due to differences in species or functional group richness (i.e. loss or gain of species or functional groups). (DOCX) [file pone.0158170.s004.docx]

**S1 Table. Components of dissimilarity between bat assemblages from ten study sites across Bolivia. Components are: βrepl = dissimilarity due to replacement of species or functional groups and βrich = dissimilarity due to differences in species or functional group richness (i.e. loss or gain of species or functional groups).**

| **Functional dissimilarity components** | | | | | | | | | |
| --- | --- | --- | --- | --- | --- | --- | --- | --- | --- |
| **β_repl_** | Yungas Alto | Yungas Medio | Pie de Monte Vargas | Pie de Monte Flores | Pie de Monte  Teran | Amazonia Ichilo | Acre Madre de Dios | Sabana Inundable | Chaco |
| Yungas Medio | 0.02 |  |  |  |  |  |  |  |  |
| Pie de Monte Vargas | 0.00 | 0.00 |  |  |  |  |  |  |  |
| Pie de Monte Flores | 0.05 | 0.07 | 0.03 |  |  |  |  |  |  |
| Pie de Monte Teran | 0.08 | 0.03 | 0.00 | 0.03 |  |  |  |  |  |
| Amazonia Ichilo | 0.13 | 0.03 | 0.01 | 0.03 | 0.03 |  |  |  |  |
| Acre Madre de Dios | 0.19 | 0.54 | 0.26 | 0.62 | 0.36 | 0.31 |  |  |  |
| Sabana Inundable | 0.07 | 0.35 | 0.18 | 0.56 | 0.29 | 0.25 | 0.61 |  |  |
| Chaco | 0.65 | 0.09 | 0.05 | 0.17 | 0.28 | 0.41 | 0.21 | 0.10 |  |
| Cerrado | 0.10 | 0.06 | 0.02 | 0.12 | 0.09 | 0.04 | 0.36 | 0.26 | 0.20 |
| **βrich** | Yungas Alto | Yungas Medio | Pie de Monte Vargas | Pie de Monte Flores | Pie de Monte Teran | Amazonia Ichilo | Acre Madre de Dios | Sabana Inundable | Chaco |
| Yungas Medio | 0.86 |  |  |  |  |  |  |  |  |
| Pie de Monte Vargas | 0.91 | 0.23 |  |  |  |  |  |  |  |
| Pie de Monte Flores | 0.66 | 0.47 | 0.63 |  |  |  |  |  |  |
| Pie de Monte Teran | 0.50 | 0.63 | 0.75 | 0.23 |  |  |  |  |  |
| Amazonia Ichilo | 0.39 | 0.71 | 0.81 | 0.35 | 0.14 |  |  |  |  |
| Acre Madre de Dios | 0.74 | 0.32 | 0.52 | 0.17 | 0.39 | 0.49 |  |  |  |
| Sabana Inundable | 0.67 | 0.45 | 0.62 | 0.02 | 0.25 | 0.37 | 0.15 |  |  |
| Chaco | 0.16 | 0.81 | 0.88 | 0.56 | 0.37 | 0.25 | 0.66 | 0.57 |  |
| Cerrado | 0.53 | 0.61 | 0.74 | 0.20 | 0.04 | 0.17 | 0.35 | 0.22 | 0.40 |
| **S1 Table. Cont.** | |  |  |  |  |  |  |  |  |
| **Taxonomic dissimilarity components** | | | | | | | | | |
| **βrepl** | Yungas Alto | Yungas Medio | Pie de Monte Vargas | Pie de Monte Flores | Pie de Monte Teran | Amazonia Ichilo | Acre Madre de Dios | Sabana Inundable | Chaco |
| Yungas Medio | 0.11 |  |  |  |  |  |  |  |  |
| Pie de Monte Vargas | 0.07 | 0.55 |  |  |  |  |  |  |  |
| Pie de Monte Flores | 0.28 | 0.22 | 0.07 |  |  |  |  |  |  |
| Pie de Monte Teran | 0.46 | 0.23 | 0.06 | 0.21 |  |  |  |  |  |
| Amazonia Ichilo | 0.58 | 0.14 | 0.03 | 0.13 | 0.45 |  |  |  |  |
| Acre Madre de Dios | 0.25 | 0.66 | 0.32 | 0.69 | 0.44 | 0.32 |  |  |  |
| Sabana Inundable | 0.26 | 0.49 | 0.26 | 0.75 | 0.52 | 0.46 | 0.75 |  |  |
| Chaco | 0.78 | 0.16 | 0.08 | 0.24 | 0.44 | 0.63 | 0.24 | 0.12 |  |
| Cerrado | 0.47 | 0.23 | 0.08 | 0.28 | 0.58 | 0.35 | 0.49 | 0.59 | 0.45 |
| **βrich** | Yungas Alto | Yungas Medio | Pie de Monte Vargas | Pie de Monte Flores | Pie de Monte Teran | Amazonia Ichilo | Acre Madre de Dios | Sabana Inundable | Chaco |
| Yungas Medio | 0.86 |  |  |  |  |  |  |  |  |
| Pie de Monte Vargas | 0.91 | 0.23 |  |  |  |  |  |  |  |
| Pie de Monte Flores | 0.66 | 0.47 | 0.63 |  |  |  |  |  |  |
| Pie de Monte Teran | 0.50 | 0.63 | 0.75 | 0.23 |  |  |  |  |  |
| Amazonia Ichilo | 0.39 | 0.71 | 0.81 | 0.35 | 0.14 |  |  |  |  |
| Acre Madre de Dios | 0.74 | 0.32 | 0.52 | 0.17 | 0.39 | 0.49 |  |  |  |
| Sabana Inundable | 0.67 | 0.45 | 0.62 | 0.02 | 0.25 | 0.37 | 0.15 |  |  |
| Chaco | 0.16 | 0.81 | 0.88 | 0.56 | 0.37 | 0.25 | 0.66 | 0.57 |  |
| Cerrado | 0.53 | 0.61 | 0.74 | 0.20 | 0.04 | 0.17 | 0.35 | 0.22 | 0.40 |
| **S1 Table. Cont.** | |  |  |  |  |  |  |  |  |
| **Phylogenetic dissimilarity components** | | | | | | | | | |
| **βrepl** | Yungas Alto | Yungas Medio | Pie de Monte Vargas | Pie de Monte Flores | Pie de Monte Teran | Amazonia Ichilo | Acre Madre de Dios | Sabana Inundable | Chaco |
| Yungas Medio | 0.04 |  |  |  |  |  |  |  |  |
| Pie de Monte Vargas | 0.02 | 0.26 |  |  |  |  |  |  |  |
| Pie de Monte Flores | 0.10 | 0.07 | 0.02 |  |  |  |  |  |  |
| Pie de Monte Teran | 0.16 | 0.09 | 0.02 | 0.09 |  |  |  |  |  |
| Amazonia Ichilo | 0.23 | 0.05 | 0.01 | 0.04 | 0.14 |  |  |  |  |
| Acre Madre de Dios | 0.11 | 0.37 | 0.18 | 0.37 | 0.20 | 0.15 |  |  |  |
| Sabana Inundable | 0.08 | 0.18 | 0.11 | 0.33 | 0.24 | 0.21 | 0.43 |  |  |
| Chaco | 0.35 | 0.05 | 0.02 | 0.08 | 0.18 | 0.29 | 0.10 | 0.04 |  |
| Cerrado | 0.15 | 0.08 | 0.02 | 0.10 | 0.20 | 0.12 | 0.24 | 0.28 | 0.19 |
| **βrich** | **Yungas Alto** | **Yungas Medio** | **Pie de Monte Vargas** | **Pie de Monte Flores** | **Pie de Monte Teran** | **Amazonia Ichilo** | **Acre Madre de Dios** | **Sabana Inundable** | **Chaco** |
| Yungas Medio | 0.86 |  |  |  |  |  |  |  |  |
| Pie de Monte Vargas | 0.91 | 0.23 |  |  |  |  |  |  |  |
| Pie de Monte Flores | 0.66 | 0.47 | 0.63 |  |  |  |  |  |  |
| Pie de Monte Teran | 0.50 | 0.63 | 0.75 | 0.23 |  |  |  |  |  |
| Amazonia Ichilo | 0.39 | 0.71 | 0.81 | 0.35 | 0.14 |  |  |  |  |
| Acre Madre de Dios | 0.74 | 0.32 | 0.52 | 0.17 | 0.39 | 0.49 |  |  |  |
| Sabana Inundable | 0.67 | 0.45 | 0.62 | 0.02 | 0.25 | 0.37 | 0.15 |  |  |
| Chaco | 0.16 | 0.81 | 0.88 | 0.56 | 0.37 | 0.25 | 0.66 | 0.57 |  |
| Cerrado | 0.53 | 0.61 | 0.74 | 0.20 | 0.04 | 0.17 | 0.35 | 0.22 | 0.40 |
